# Health sector solidarity: a core European value but with broadly varying content

**DOI:** 10.1186/2045-4015-4-5

**Published:** 2015-04-17

**Authors:** Richard B Saltman

**Affiliations:** Department of Health Policy and Management, Rollins School of Public Health, Emory University, Atlanta, GA USA

**Keywords:** Solidarity, Social health insurance, European values, Austerity

## Abstract

Although the concept of solidarity sits at the center of many European health sector debates, the specific groups eligible for coverage, the financing arrangements, and the range of services and benefits that, together, compose the operational content of solidarity have all changed considerably over time. In prior economic periods, solidarity covered considerably fewer services or groups of the population than it does today. As economic and political circumstances changed, the content of solidarity changed with them. Recent examples of these shifts are illustrated through a discussion of health reforms in Netherlands, Germany and also Israel (although not in Europe, the Israeli health system is similar in structure to European social health insurance systems).

This article suggests that changed economic circumstances in Europe since the onset of the 2008 financial crisis may lead to re-configuring the scope and content of services covered by solidarity in many European health systems. A key issue for policymakers will be protecting vulnerable populations as this re-design occurs.

## Introduction

The idea of solidarity has been at the center of European health policy at least since the end of World War Two [1]. Whether officially termed solidarity (as in social health insurance systems and also tax-funded systems on the Continent) or phrased as its conceptual near-equivalent “equity” (UK) or “equality” (Nordic countries) in Northern European tax-funded health systems, solidarity has long been acknowledged as a core value in European health policy debate. Any policy proposal that seeks to change coverage or access components within a country’s health sector is assessed in terms of its likely impact on solidarity, and a major element of its political success or failure often reflects that impact.

In most of these national policy debates, participants rarely define what is meant by that term, “solidarity”. Its content is assumed to be understood by all, and is normally not specifically defined in public discourse or, in some countries, even in its main health sector legislation. Such an explicit definition has seemed unnecessary, as in most cases all sides in the political debate are committed to this core value, and reflexively re-state their commitment as they go forward to deal with the specific policy issue under consideration.

The concept of solidarity used in European public debate has both aspirational (philosophical) and practical (operational) dimensions, sometimes combined in the same statement. Historically, the concept of solidarity in Europe has referred to “communities of mutual recognition” [1]. These typically grew out from personal (family) to communal (churches) to occupational (guilds, unions) and finally to national (political associations and parties) when the state stepped in as the financial regulator and guarantor [2]. Philosophically, a variety of attributes were assigned to solidarity: reciprocity (asymetrical for the needy); social cohesion; altruism and fellowship; citizenship duties; universal brotherhood; political and/or social justice [3]. Operationally, although the original self-governing institutions of solidarity were retained when the state took control, in practice decisions were “lifted out of the context of mutual recognition” to become incorporated into the state’s administrative apparatus [1].

In current practice, the term solidarity has a number of different meanings to the range of different political actors who invoke it. To some, solidarity means that every individual regardless of income or social standing has the same services delivered by the same health care providers and with the same clinical outcome. To others, solidarity is more focused on equal process - particularly access to care - rather than equal clinical or health status results. To yet a third group, solidarity refers to shared financial sacrifice – guaranteeing that funding responsibilities are acceptably distributed across a particular group or population. In actual practice, the particular health service content that solidarity covers - “who gets what, when, and how” to apply Lasswell’s famous definition of politics - has varied quite considerably, depending on the political and economic era, on the country’s norms and values, and on the particular structure of the country’s health-related institutions [2]. These operational differences typically are subsumed within the language of solidarity as the philosophical “touchstone” of what is viewed as a core normative value.

This article explores the changing meaning and content of solidarity in two different European social health insurance systems and in the similarly structured social health insurance system in Israel. The next two sections take a broadly historical perspective, reviewing the specific practical content of solidarity as it has evolved up to the present period. Most (although not all) of these changes have served to increase the overall services and population groups covered by the collectively financed system. The subsequent section then considers the implications of Europe’s slow/no growth economies for future health reform and the types of measures that have been or are being considered to revise what is covered by solidarity in their health system, this time mostly toward reducing collectively covered coverage and services. The paper concludes with a short assessment of the implications of this reviewed experience for the concept of solidarity generally.

### Historical evolution of solidarity’s content

The first practitioners of solidarity were the friendly societies formed by craft workers in the Thirteenth Century in England [4]. Over the next several centuries the concept continued to grow in various cooperative and not-for-profit private settings, becoming particularly associated with industrial workers’ mutual societies and, in the latter part of the 19th Century, the union movement [2].

By the end of the 19th Century, as more traditionalist politicians were looking for tools to blunt the growing political power of Marxist and/or Lassallean socialist movements among factory workers, the concept of solidarity and its collectively paid set of cross-subsidized health benefits took more comprehensive shape. Through this political process, the concept – and practical benefit content – of solidarity as a central function and objective of collective health insurance migrated from its original source in voluntary organizations in civil society to becoming a shared responsibility between these private sector organizations and the State, with a set of state-imposed statutory responsibilities backed up by formal State regulation and supervision [2].

During this period of growth and expansion, the individuals and the range of services covered were continuously evolving, driven by improving industrial productivity that made possible a rising level of funding from both public and private sources, as well as a rapidly developing level of medical technology. Cultural norms and values also played an important role in shaping the specific services that solidarity should cover. In earlier times, for example, an important component of workers mutual insurance was a death benefit, to ensure that a covered member could afford a proper Christian burial and thus not suffer the ignominy of a pauper’s grave. Even in current times, social health insurance in Germanic countries still covers culturally (but not necessarily medically) desirable “kur” or medical spa treatments.

As the above observations suggest, solidarity has historically been a broadly flexible concept, adapting to and reflecting the changing historical, economic, political and social environment around it. In this sense, solidarity has never been something with a fixed content or an immutable character. Moreover, when one considers that the core modern meaning of solidarity – equal treatment for all social groups (including elderly, low-income, immigrants, disabled) which now similarly exist in Northern European tax-based systems defined as norms of “equity” (UK) or “equality” (Nordic countries) – it seems clear that this central value sitting at the core of European health systems is not a fixed notion but rather a flexible concept with a multi-faceted, contingent content.

### Country examples of differential/variable solidarity

The wide-ranging health sector services and benefits that compose the real-world notion of solidarity is immediately apparent in the broad distribution of citizen and service coverage that different health systems label as “solidaristic.” This is noticeable both in the varying groups of citizens covered by specific national social insurance programs, and also in the varying services and benefits that these programs cover.

### Netherlands

Concerning coverage, consider the Netherlands prior to 2006. The statutory health insurance system for regular curative medical care (there was a separate social insurance system for “exceptional” or long term care that covered 100% of the population) only covered about 63% of all citizens [5]. Those whose income was (or grew to be) above the ceiling threshold (32,500 Euros in 2004) were required by law to leave the statutory system This meant, on the one hand, that these higher-income individuals were required to seek private commercial health insurance, which set its premiums based on individual experience (eg personal health-status and family history) rather than community experience (eg collective) costs. This could be particularly expensive for workers in their 40s and 50s for whom a small raise in income could push them over the income boundary into a much more expensive insurance category. Moreover, forcing out higher income earners meant that the “solidarity” of the statutory system was only a solidarity of low and middle income earners – those who earned higher incomes did not have to (indeed could not – at least legally) participate in the collective risk pool. Thus in pre-2006 Netherlands, solidarity was not universal in nature. Yet Dutch scholars writing in this pre-2006 period regularly hailed the normative achievement created by the solidaristic dimensions of their health insurance system [6].

It is equally instructive to consider what solidarity now means in the Netherlands, following the 2006 health insurance reform [7]. In the re-structured arrangement, 100% of citizens must purchase regular medical insurance through the statutory system (the so-called “mandate”) from any insurance company they desire that operates in the Netherlands [7]. So in this first respect, the post-2006 Dutch health insurance system dramatically increased solidarity, in that all higher income citizens were for the first time required to be in the same insurance pool as their lower income neighbors for regular medical care (they already were for the ABWZ for Exceptional and Long Term Care expenses), and to cross-subsidize the cost of coverage for lower-income citizens.

Yet the post-2006 insurance system had a new feature that split the purchase of insurance coverage into two linked segments (a third segment with especially challenging solidarity characteristics – a no-use rebate of 255 euros, which had to be paid by covered individuals up front - was dropped in 2008). One of the two remaining segments in the post-2006 insurance model required that the employer’s 50% share of the total premium cost must continue to be channeled through a single national state-run fund [8]. This fund would allocate a risk adjusted payment for each covered individual to whatever (private not-for-profit or for-profit) insurer the individual selected. Quite differently, however, the second, individually paid portion of the mandatory insurance premium – labelled the “nominal premium” – was to be used to purchase a second part of the individual’s insurance policy with that same selected company, with most individuals paying the funds directly him or herself from his/her own private funds (some low income families received a state-paid “healthcare allowance” until these subsidies were cut). Moreover, to encourage bargaining that could generate efficiencies from the private insurance companies, individuals could reduce their premiums for this second part of their regular medical insurance by up to 10% by banding together with other Dutch citizens to negotiate volume discounts either via a real organization (for example, an employee group or a patient association ) or via a virtual organization formed over the internet, typically solely for the purpose of negotiating just such a volume discount [7].

Thus, while the first part of the post-2006 regular medical insurance considerably increased solidarity by mandating that all citizens must join the same risk pool, the second part of that insurance arrangement considerably reduced solidarity by enabling citizens to separate themselves into smaller market-based groupings that could negotiate a better deal for themselves than the one that other citizens might have. Thus the post-2006 system both increased risk sharing in the employer paid segment but decreased risk sharing in order to encourage market efficiencies in the individual-paid “nominal premium” segment.

Moreover, in a separate part of the post-2006 regular medical insurance, the state now pays the entire insurance premium for all children, further muddying the solidarity waters in that this initiative reduced costs for both higher as well as lower income families. Overall, it is unclear whether the post-2006 Dutch insurance plan actually did increase solidarity as much as proponents claim.

Two additional elements of the new system further complicate the solidarity characteristics of the Dutch post-2006 insurance picture. The first is an additional State bundled payments scheme for those who suffer from what are termed “listed diseases” (eg diabetes, chronic heart failure, COPD) [7]. These additional payments, made through the risk adjustment mechanism to the individual’s selected insurer, are intended to compensate the private insurance company for the additional cost of that patient’s condition, and thus to relieve the insurer of most financial risk and, one might note, to relieve that individual’s fellow insurees of the need to cross-subsidize his/her care – thus simultaneously increasing (by paying more for sicker individuals) and reducing (by taking financial pressure off the second “nominal premium” payments) solidarity via this extra disease-based payment. The result of these extra payments has been - just as the Dutch government had hoped – that individuals who suffered from these listed diseases have been welcomed by the private insurers – who indeed sometimes actually advertised for individuals with these conditions to select their insurance company. This result is exactly the reverse of the adverse selection often found in private insurance markets, and can be considered on balance an improvement in the solidarity characteristics of the post 2006 Dutch health insurance system.

Conversely, however, the second additional dimension concerns the purchase of supplemental medical insurance, which had been widely purchased by those with mandatory social health insurance in the pre-2006 system, and which continues to be sold by private insurers in the post-2006 arrangement. This supplemental insurance pays for additional private services such as physiotherapy and dental care, as well as quicker care for some outpatient medical services [9]. Thus this second dimension of the post-2006 system continued what had been – prior to 2006 – something that also had reduced solidarity, in that the lowest income citizens typically could not afford to buy it.

### Germany

Moving beyond the Netherlands, a similar profile of collective versus individual risk within a solidaristic health system also can be observed in Germany’s social health insurance system. In Germany for regular curative services (since 1996 Germany also has a separate social insurance fund for long term care), a similar (although higher) income ceiling still exists [10]. However in the German variation, there are two important differences from the pre-2006 approach of the Netherlands.

First, those whose income rises over the statutory ceiling can, if they choose, remain in the statutory, collectively based insurance system (with the interesting caveat that, if they ever leave the statutory system, they cannot return, even if their income should fall below the statutory ceiling at some point in the future, although exemptions exist for those who move from self-employed to employed or who leave their job and are eligible through a spouse for statutory family insurance).

Second, unlike the Netherlands, this ability of higher income earners to leave the compulsory system – and thus to break solidarity with lower income wage earners – is still in place, as is the exemption that State civil servants have long had to receive separate indemnity based health insurance [11]. Interestingly, this break in solidarity based on income has been continued despite Germany putting in place a major health insurance reform in 2009 that centralized the collection of all health insurance premiums into a single state-run pool that, as in the Netherlands, then risk adjusts the amounts paid out for each covered person to their choice of health insurance fund [12].

Thus in two of the most prominent social health insurance examples of countries where “solidarity” is the widely proclaimed basis of their health system, the requirement for who has (or is allowed) to share in the collectivist insurance system, and thus how solidarity is put into place, has long varied and indeed varies considerably still in 2014.

### Israel

A third country example is Israel, which, although not territorially in Europe, has a social health insurance system that resembles both Dutch and German arrangements. Prior to 1995, solidarity was strongly defended in Israel as the central characteristic of each of the four social health insurance funds. Each of these funds was a separate not-for-profit private entity that collected premiums from its membership and in return provided primary care, hospital and ancillary health-related services. The largest fund, Kupot Holim Clalit, was closely tied to the national labor federation (Histadrut), and social health insurance premiums were collected as a portion of each member’s labor union dues. Yet because premiums were the same regardless of income, this pre-1995 Israeli system, like most traditional social health insurance systems, was mildly regressive. Moreover, the pre-1995 system was not universal, as some 5% of the Israeli population, especially among Arab Israelis, were not members of any of the four funds [13]. Thus the pre-reform arrangement had several structural dimensions that undercut rather than reinforced solidarity.

In 1995, a new funding arrangement was introduced for the health sector. Individuals pay a flat-rate income tax of 3.1% on wages up to half of the average national wage and 4.8% on income beyond that level. Income above five times the national wage is not subject to this health tax. There also are exemptions and discounts for various groups such as pensioners and recipients of income maintenance allowances. This health tax raised 25% of total health expenditures, and was supplemented by general state revenues which made up some 46% of total health sector funding [13]. All these public funds were collected in a central national pool, which then allocated these funds on a per capita risk adjusted basis to whichever of the four funds a citizen chose to join. This state-centralized, citizen-based funding model covered all Israeli residents, improving solidarity considerably not only among Jewish Israelis but also by incorporating a significant number of Arab Israelis who had not been part of the prior system [14]. While 75% of Israelis also buy supplemental insurance (typically from their health fund), these additional insurances are since 1998 required to be community rated so as to reduce their impact on higher risk individuals and thus add a solidarity increasing dimension to an otherwise solidarity decreasing mechanism.

This new post-1996 system, however, still presents several dilemmas regarding the level of solidarity it generated. One dilemma is that the four funds (now called health rather than sickness funds) continue to sell supplemental insurance to their membership, covering additional clinical services that are not covered by the standard tax-paid premiums. While, as mentioned, some 75% of Israelis purchase these supplemental policies, there is a social gap as only 62% of lower income citizens can afford or choose to purchase these policies [13].

The second equity-based concern was of a different character, in that it involved what services would be covered under the tax-funded health system. Under the 1995 reform, this determination was to have been made by a 70-member council, made up of representatives of key stakeholders in the Israeli health system [14]. Among the most difficult decisions have been those involving new pharmaceuticals that, for cost or efficacy reasons, are excluded from the government-funded benefit package. Major fights occurred over a new multiple sclerosis drug, for example, which ultimately was included [15].

Eventually, decisions regarding additions to the basket of services were delegated to a committee made up of medical people, economists and public representatives, which receives an annual budget for this purpose. Of course, not all possible technologies can be added due to fiscal constraints. For example, the cancer drug Avastin was not included by the committee for treatment of colon cancer. This rejection produced a public hunger strike by some potential patients, and while additional budget funding was allocated to the committee, ultimately the drug’s exclusion from the benefits package was confirmed [16]. Even when the decision process is democratic and transparent, however, each time a drug is excluded from the publicly funded package, there is a reduction in solidarity with those patients who would have benefited from its inclusion.

Israel’s experience with new drug inclusion mirrors a similar pattern that has occurred in England, where the National Institute for Clinical Excellence has made a number of cost and efficacy based exclusion decisions concerning the formulary provided by the National Health Service which were contested by patients and, in some cases (Herceptin) overturned by national political decision-makers [17]. In 2013 decisions by NICE about new drug adoptions were changed from mandatory to advisory in nature for NHS institutions [18].

Thus in Israel as in Netherlands and Germany, an historical pattern of strong but also somewhat erratic solidarity became more comprehensive as the result of a recent national health reform, yet several other dimensions of the reformed payment structure (eg the continued existence of supplemental health insurance and the exclusion of certain life-extending drugs from the benefit package) simultaneously served to reduce that new level of solidarity.

### Additional dimensions

The three case examples given here provide only the big-picture structural dimensions of how these three social health insurance systems differ in their practical content from the core values assumed to inhere to solidarity. One could deepen this picture by introducing concerns about pensioners, the disabled, the self-employed, university students, immigrants, and other vulnerable populations whose care is funded under a wide variety of special and not always normatively similar arrangements within these “solidaristic” systems [11]. One also could expand this discussion by reviewing the long history of change in services, benefits, and different population groups, and the differential treatment received by individual citizens in different geographic localities, found in the tax-funded but solidarity based Mediterranean health systems (Italy, Spain and Portugal) as well as in the tax-funded equity or equality based health care systems in Northern Europe including England and the four European Nordic countries (Sweden, Finland, Norway, and Denmark). There are, in sum, many different and complex dimensions in how services and benefits have been and still are allocated within countries whose health systems consider themselves to be based on the core European value of solidarity.

### Present-day health sector consequences of inadequate economic growth

The impact of the 2008 financial crisis remains strong in European health care systems. While economic growth has begun to return to some countries in the EU, its pace is painfully slow, and its future is not at all secure [19]. In the Netherlands, for example, first quarter 2014 GDP fell by 1.4%, while for the same quarter in Finland, the economy slipped back into contraction for the third time in 5 years [20]. In the UK, although the economy has begun to grow, total annual GDP in the first quarter of 2014 is still below what it was in 2008 [21]. Confirming continued underperformance, on 4 November 2014 the European Commission (the executive arm of the European Union) cut its forecast through to 2016 for the 18 country Eurozone from 1.7% to 1.1% GDP growth – a level which is just barely more than recession level [22].

This now seven-year lack of substantial growth in national economies has restricted the amount of new and even previous funding that is available for publicly funded health sector activities. In England, for example, the recently installed head of the NHS, Simon Stevens, was quoted in the Financial Times on 31 March 2014 as saying that “the traditional model of healthcare will have to change as the NHS faces the ‘most sustained budget crunch in its 66-year history’” [23]. A King’s Fund report documented that the NHS in 2015 faces a 30 billion pound shortfall in a total budget of some 100 billion pounds [24]. The Financial Times reported that 5 of 6 hospital trusts faces a projected fiscal shortfall in that same NHS budget [25].

In the Netherlands, the King stated on 17 September 2013 in his speech opening the Dutch Parliament’s fall session that the prior generous welfare state model was no longer affordable [26]. One consequence for the health sector in the Dutch case – as in the English as well – is that discussions have occurred about new patient payments that might be necessary. As one example, the rising number of elderly in need of custodial and long term care has triggered discussions in both countries about the possibility that more elderly middle class patients may be required to sell their homes in order to pay for nursing home services [27].

A further insight into the current post-recession environment of 2014 can be gleaned from current statistics collected by the World Health Organization’s Regional Office for Europe regarding out-of-pocket co-payments as a percentage of total health sector finances in different European Union countries [28]. These largely un-solidaristic payments (although some co-payments for drugs may be tied to age or health status) range from 5.9% in France and 9.9% in England to 20.2% in Italy, 20.3% in Spain, and 18.4% in Estonia up to 37.4% in Latvia [28]. While all these EU health systems would each still say that they continue to honor the European value of solidarity in the finance of their health care system, the range in out-of-pocket payments among these countries currently differs by as much as seven-fold.

To be sure, there are some countries (Germany, Sweden, Norway, Israel) where economic growth has continued if in measured rather than robust terms. In these countries, the health coverage debate about the need to re-consider the content of the publicly funded health sector package typically has had more of a future planning than an immediate management character. However the slow rate of underlying economic development in developed countries since 2008 (and indeed for two decades prior) will ultimately affect all European health systems, as strong economic growth and the consequent wealth production continues to consolidate in Asian Rim countries not in the West [29].

### Some implications for the concept of solidarity

The above discussion suggests several points about the character of solidarity as it has been structured in the European health sector. The first is that solidarity has traditionally been a flexible concept, varying considerably across countries and across historical periods in the types of content it covers and for whom. Thus assertions that the present level of solidarity in the health sector cannot be altered without undermining the entire concept and breaking faith with the citizenry do not comport with past practice.

Similarly, the contention that governments should not consider reductions in what services and benefits are covered in a solidaristic system is inherently more political than factual. This is particularly true if the financial sustainability or even the long-term survival of existing health systems in some countries may be at stake. As the examples above highlight, in practice national governments have periodically changed both the content of their solidaristic health systems and the population groups that have membership in these systems. Given past practice, then, what would be new and unusual in the current situation would be if governments no longer tailored their health system funding and benefit structures to the available level of public sector resources consistent with prudent fiscal management.

To be certain, most European policymakers themselves do not want to reduce services and coverage, they want to protect vulnerable groups, they want to expand coverage for newly growing groups such as the chronically ill elderly, and there are numerous health policy arguments that support those preferences [30]. However the argument that an expansive health policy approach is an inherent requirement of health sector solidarity as a core European value is inconsistent with past experience. Past practice clearly indicates that governments can and regularly do change the social contract that underlies health sector solidarity, and that further changes to that basic contract can in some circumstances be in keeping with good governance rather than necessarily reflecting a breach of that stewardship role [31].

This is not to argue that reductions in service coverage or collective payment are good things that national policymakers should seek for their own sake. Rather, it is to validate the types of changes recently seen in a number of European health systems since the onset of the 2008 financial difficulties, and to recognize that changes that reduce as well as expand the degree of coverage and benefits reflect the principle of solidarity as it has been practiced previously.

An important question can be raised about what an appropriate new blend of mandatory social vs. supplemental insurances might look like in response to fiscal stringency, and the degree to which the distribution of specific costs and/or services between different types of coverage might reasonably be changed. Further, as with supplemental insurance in Israel, where governmental regulation requires that such policies be written on a community rating basis, future shifts in the balance of public mandatory and supplemental insurance could potentially be structured to provide more rather than less coverage for socially vulnerable groups. The challenge presented by ongoing economic constraints will be to shape necessary reductions in solidarity in operational terms in a manner that does the least harm to present day health and social objectives of solidarity in philosophical and normative terms.

A second point is that ongoing rapid developments in the health sector including genetic decoding, personalized medicine, bio-engineered pharmaceuticals, miniaturized diagnostic and treatment methodologies, and targeted wireless information technology will further raise the international standard for how clinical medicine will be practiced in the near future, and, in many cases, will raise the cost of operating high quality health systems. However, reflecting the ongoing shortage of new public funds and personnel, these new clinical developments suggest that the international standard will likely be increasing at the same time as at least some European health systems will already be making reductions in the range and content of existing clinical services and benefits that they provide.

These constraints on publicly funded benefit packages will be taking place, further, not only as the international standard of clinical care that their citizens expect to receive increases, but at a time when some public health proponents in Europe believe there should be expensive new commitments of public funding to support long-term “upstream” preventive health interventions in housing, education, transportation, and employment in order to rectify inequities in the “social determinants of health.” [32, 33]. These competing pressures on restricted public resources, taken together, would appear to suggest that the operational content of solidarity between and perhaps even within countries in Europe could continue to vary considerably over the next period of years.

## Authors’ information

Richard B. Saltman is Professor of Health Policy and Management at the Emory University School of Public Health in Atlanta, Georgia. He was a co-founder of the European Observatory on Health Systems and Policies in Brussels in 1998, and is currently Associate Director of Research Policy. He is also a co-founder and co-director of the Swedish Forum for Health Policy in Stockholm. He holds a doctorate in political science from Stanford University.
